# Indicators of breast cancer severity and appropriateness of surgery based on hospital administrative data in the Lazio Region, Italy

**DOI:** 10.1186/1471-2458-6-25

**Published:** 2006-02-07

**Authors:** Patrizia Schifano, Paolo Papini, Nera Agabiti, Marina Scarinci, Piero Borgia, Carlo A Perucci

**Affiliations:** 1 Department of Epidemiology, Health Local Unit RME, Rome, Italy; 2 Agency of Public Health, Lazio Region, Italy; 3 Department of Preventive Medicine, Health Local Unit RMD, Rome, Italy

## Abstract

**Background:**

Administrative data can serve as an easily available source for epidemiological and evaluation studies. The aim of this study is to evaluate the use of hospital administrative data to determine breast cancer severity and the appropriateness of surgical treatment.

**Methods:**

the study population consisted of 398 patients randomly selected from a cohort of women hospitalized for first-time breast cancer surgery in the Lazio Region, Italy. Tumor severity was defined in three different ways: 1) tumor size; 2) clinical stage (TNM); 3) severity indicator based on HIS data (SI). Sensitivity, specificity, and positive predictive value (PPV) of the severity indicator in evaluating appropriateness of surgery were calculated. The accuracy of HIS data was measured using Kappa statistic.

**Results:**

Most of 387 cases were classified as T1 and T2 (tumor size), more than 70% were in stage I or II and the SI classified 60% of cases in medium-low category. Variation from guidelines indications identified under and over treatments. The accuracy of the SI to predict under-treatment was relatively good (58% of all procedures classified as under-treatment using pT where also classified as such using SI), and even greater predicting over-treatment (88.2% of all procedures classified as over treatment using pT where also classified as such using SI). Agreement between clinical chart and hospital discharge reports was K = 0.35.

**Conclusion:**

Our findings suggest that administrative data need to be used with caution when evaluating surgical appropriateness, mainly because of the limited ability of SI to predict tumor size and the questionable quality of HIS data as observed in other studies.

## Background

Breast cancer is a major cause of cancer death among women in the region of Lazio (central Italy, including Rome), and in all of Italy [1,2]. Several randomized clinical studies have shown that simple mastectomy is as effective as more radical treatment (Halsted's radical mastectomy) in terms of survival [3,4]. Although national and international guidelines list treatment protocols specific to the tumor's stage [5,6], the degree of adherence to these guidelines in Italy is not known, and in recent years the surgical techniques for breast cancer used in different Italian hospitals have varied widely [7,8]. Clinical stage is the principal determinant of surgical treatment; specifically, there has been an under-use of conservative procedures with stage I and II tumors [9]. Stage I (up to 2 cm in diameter) and stage II tumors (more than 2 cm in diameter) can be treated with conservative surgical intervention (lymphoadenectomy and quadrantectomy) as effectively as with radical mastectomy [10,11].

In Italy, regional hospital information systems (HIS) contain information on all hospital admissions. They are a valuable source of data for verifying the quality and appropriateness of health procedures and/or in evaluating the economic impact and frequency of use of new technology [12,13]. Current literature which uses HIS data to evaluate breast cancer surgery is limited to geographic and temporal variability of various surgical procedures and their possible determinants [14,15]. A recent study conducted in Australia described geographical variations in surgical treatments offered to women with breast cancer [16], while an American study showed that conservative treatment is most common among young women, and more frequently performed in university hospitals [17]. Data analyses from the London Tumor Registry showed noticeable differences in therapeutic procedures in the 42 hospitals involved in the 1980's study [18]. Age and education level appear to play important roles in the type of surgical intervention offered [19]. A direct relationship was observed between volume of hospital activity and adherence to guidelines [20,21]. Various studies in Italy also have documented levels of inappropriateness according to hospital and patient characteristics [7,22]. In Lazio, age, tumor severity and hospital volume were associated with the use of conservative therapy [8].

HIS data have important limitations for breast cancer surgery, mainly because they do not contain information on tumor stage, available only in medical charts, the crucial indicator for determining appropriate treatment, and more generally, because administrative data completeness and accuracy have not been established.

Despite the absence of tumour size in the HIS many authors used the combinations of primary and secondary ICD9-CM diagnosis to estimate breast cancer severity [21,23].

The aims of this study are to: 1) validate a breast cancer severity indicator (SI) calculated from primary and secondary diagnoses (ICD-9-CM codes) from the HIS; 2) verify the differences in the SI, tumor stage, and tumor size in evaluating appropriateness of breast cancer surgery, and, 3) verify the accuracy and completeness of the HIS in recording breast cancer diagnoses and surgical procedures.

## Methods

### Study population

A sample of 398 hospital admissions were selected from a Hospital Information System (HIS) based cohort of 4823 women with a principal diagnosis of breast cancer (ICD-9-CM: 174.0–174.9) or of breast cancer *in situ *(ICD-9-CM: 233.0), who were residents of the Lazio region, (region of central Italy, including Rome, about 5 millions inhabitants and 180 hospitals). and had their first surgical intervention (ICD-9-CM: 85.20–85.25; 85.41–85.48) between January 1997 and June 1998 in any one of the regional hospitals. Patients were excluded if they had previous hospital admissions for malignant breast cancer other than for diagnostic purposes.

### Lazio region HIS

Since 1994 the HIS [24] has archived the data from hospital discharge records (HDR) of all Lazio regional hospitals. The HDR summarizes information from clinical charts regarding type of discharge, primary diagnosis, up to three secondary diagnoses and four surgical, diagnostic or therapeutic procedures, codified according to the International Classification of Disease and Causes of Death (ICD-9, 9^a ^revision) [25].

### Data source

For each hospital admission a photocopy of the original clinical documents was requested. Three physicians, who were also experts in classification systems and disease codifying, re-examined the clinical charts and reported relevant information onto a two part *ad hoc *schedule. The first part requested both non-coded diagnoses and ICD-9-CM coding from the HDR; the second part contained clinical and anatomy-pathology parameters, including tumor size (pT), and diagnostic results to calculate the stage of the tumor (TNM) [5].

### Definition of variables

A) Indicators of tumor severity

### Tumor size

Size (pT) is classified into the following categories according to pathology reports included in clinical charts: Tis for carcinoma in situ/Paget, T0 for "no evidence" of a local lesion; T1 for tumors up to 2 cm in diameter; T2 for tumors from 2 to 5 cm; T3 for tumors greater than 5 cm, and T4 for tumors that have spread into surrounding tissue [5].

### TNM staging system

The TNM stage was determined from clinical charts; this defines five stages of breast cancer severity (from 0 to IV) based on the size, but also on other characteristics of the tumor, positive lymph nodes, and other sites of metastasis [5]. Consequently, some categories correspond to tumor size, while others do not. For example, a tumor up to 2 cm in size (T1) with positive lymph-nodes is equivalent to a bigger tumor (>T1) without positive lymph nodes.

### Severity indicator (SI) based on the HIS diagnosis

The severity indicator we used is a modified version of the indicator defined by Kahn et al [23], based on specific ICD9 codes for breast cancer. It allows breast cancer cases to be classified into 4 levels of clinical severity, from the primary and secondary diagnoses reported in the HIS (table 1). First level (I): *tumors in situ*; second level (II): *localized tumors*; third level (III): *non-localized tumors with loco-regional metastasis*; IV: *non-localized tumors with distant metastasis*. The SI groups tumors according to their principal characteristics (localized/not-localized; presence/absence of metastasis) like the TNM system, but does not include tumour size(pT).

B) Indicators of intervention appropriateness

We referred to treatment Italian guidelines for breast cancer [5], which indicate

- conservative surgery for tumors up to 2 cm (for at least 80% of cases);

- Halsted mastectomy only for tumors that have infiltration of the thoracic wall;

- radical excision of auxiliary lymph nodes (or possibly the search for sentinel lymph nodes in accredited hospitals) in all cases except for tumors *in situ.*

According to the above criteria and to the review of clinical charts interventions which varied from these indications were classified as "under-treatments" or "over- treatments". Table 2 shows the definitions of over- and under- treatment using the three aforementioned indicators of tumor severity (pT, TNM, SI).

### Data analysis

The agreement between SI and TNM classifications and between SI and pT classifications (Intraclass correlation coefficient, Spearman's Rho) [26] were determined. We compared their ability to classify the appropriateness of interventions, using pT as the gold standard for defining "under-treated" and "over-treated" cases. The reason for this choice is that pT is the only information surely available to every surgeons at the moment of choosing the therapeutic intervention. Thus, sensitivity, specificity and positive predictive values (PPV) were calculated respectively for SI and TNM staging.

Lastly, we evaluated the accuracy of the data from the HIS by measuring the agreement between the SI calculated from the ad *hoc schedule *and from the clinical charts, through a weighted kappa statistic. The PPV of the HIS was also calculated.

Data were analyzed using SPSS [27].

## Results

Clinical charts were obtained for 387 of the 398 cases included in the sample (97.2 %). Sixty cases (Eleven cases according to SI and 53 according to TNM stage and pT) could not be assessed, due to unclear or incomplete charts. According to the SI indicator, most of the cases had a medium-low level of severity (about 66% in class 2). Based on the TNM, more than 70% of cases were classified stage I or II; based on pT, most cases (48.1%) were classified as T1 (Table 3).

**Figure 1:**
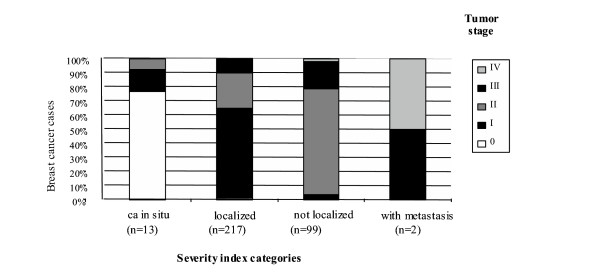
Distribution of tumor stage by severity index

A positive correlation (ρ = 0.6; p < 0.001) was observed between the severity indicators SI and TNM and the distribution of tumor stage in each category of the SI, showed in Figure 1, suggest a rather good agreement between the two classifications. However, SI did not correlate as well with pT size (ρ = 0.3 p < 0.001). In fact in class 1 of the SI 77% of tumors were size Tis and 15% were size T1, in class 2 66% were T1 and 24% T2, in class 3 37% were T1, 44% T2 and 14% T4. Because only two cases belonged to class 4 the percentage was not calculable.

Based on pT, 28 % (94/334) of cases were under-treated and 5% (17/334) were over-treated; based on TNM stage, the percentages were 35% (117/334) and 5% (18/334) and on the SI they were 25% (82/334) and 6% (19/334) respectively (Table 4). The SI and pT (gold standard) agreed relatively well in classifying "under"- and "over-treated" cases (kappa = 0.53). The agreement between the "under" and "over-treated" cases classified on the basis of the TNM and tumor size pT was very high (kappa = 0.82). The accuracy of the SI in estimating under-treatment was fair (58% of all procedures classified as under-treatment using pT where also classified as such using SI; 67.1% of all procedures classified as under-treatment using SI where classified as such according to pT), while it was quite good for over-treatment (respectively 88.2% and 78.9%). The TNM staging system showed values of 96.8% and 77.8% for under-treatment and 100% and 94.4% for over-treatment (Table 4).

Lastly, the total agreement between HIS data and clinical charts was quite low (kappa = 0.35) and the PPVs (Table 5) were 11% for class 1, 75% for class 2 and 76% for class 3. Class 1, tumors *in situ*, in particular showed very low PPVs. A misclassification was observed for tumors *in situ *by the HIS; it misclassified 8 invasive tumors as cancer *in situ*, and failed to identify 14 out of 15 of them. As a result, 67 cases were listed as "localized tumors" in the HIS but as "non-localized" on the clinical chart.

## Discussion

The study indicates that with data from hospital discharge reports it is possible to construct an indicator of breast tumor severity that performs quite well compared to clinical and histological classifications. However, it needs to be used with caution in estimating the appropriateness of surgical treatment, because although it is sufficiently thorough in estimating over-treated cases, it tends to underestimate under-treatment. The validity of the severity indicator from the HIS depends explicitly on the completeness and quality of the data.

Our proposed indicator is based on ICD-9-CM codes of breast cancer diagnoses and procedures, and uses some of the criteria proposed by Kahn et al in 1996 [23]. Since the ICD-9-CM coding does not include tumor size, their indicator is based on the assumption that there is a positive correlation between the extent of the growth (localized, loco-regional, and distant) and the size of the tumor. Consequently, the criteria were based on the level of diffusion of the tumor from the ICD-9-CM codes, which served as a proxy for tumor size, and thus for severity. Kahn et al evaluated the accuracy of ICD-9-CM codes in correctly identifying localized breast cancer, and then in documenting the percentage of lymphoadenectomies that had been performed, comparing to the data from the cancer register. The authors calculated the raw agreement of the two information sources was 82%; the sensitivity and specificity was acceptable (above 85%). Thus, the data from the HIS were considered valid in evaluating appropriateness of surgery typology according to tumor severity (localized/not localized), but not for analyzing therapeutic outcomes[23]. Our study modified Kahn's SI to better differentiate degrees of severity: we considered cancer *in situ *as a category in and of itself (otherwise included in the same category with localized tumors) and we distinguished, in the non-localized tumor category, those with loco-regional metastasis from those with distant metastasis. A similar approach is found in the study conducted by Rohan et al [21].

The correlation of the SI with TNM stages was rather good (0.6; p < 0.001), but not as good with tumor size (pT) (0.3; p < 0.001). These results suggest that the SI can be considered an adequate proxy of tumor severity, but is not as precise an indicator of tumor size. Unfortunately, tumor size is usually the only indicator available to physicians when determining treatment.

We also evaluated the possibility of using the SI to evaluate appropriateness of treatment: the SI appears to classify cases of over-treatment relatively well but underestimates under-treated cases. We found that TNM and pT classifications are better indicators of under- and over-treatment; this may be because size is a component of TNM staging. It is, however, important to note that the SI seems a conservative indicator of both over-treatment and under-treatment, minimizing the risk of inappropriately categorizing difficult cases.

In order to rule out possible errors of classification, codification or input, data registered on an *ad hoc *schedule were used instead of the original HIS information to construct the SI. Subsequently however, the completeness and accuracy of the discharge reports were assessed comparing them to clinical documents: this study was a part of a larger study on quality of data from HIS, methods and results are available on technical report published on web [28]Hospital discharge records have been shown to have problems of under-notification of health data, which greatly decrease their value to analytic studies, [29,30]. Furthermore, in our study there were a number of cases for which it was not possible to define the TNM stage due to lack of information on the clinical documents, which could have in part reduced the "validity" of our gold standard. The PPV of the HIS is overall quite low (Kappa = 0.35), mainly due to a high level of discordance in the first SI category (carcinoma *in situ*). The PPV in the other three categories comparatively is rather good.

## Conclusion

Our findings support previous literature results that hospital discharge reports are potentially utilizable to define the clinical severity of breast cancer, mainly for the purpose of public health evaluations, such as studying variations in breast cancer treatment in key demographic groups. The limited ability of SI to predict tumor size and the questionable quality of HIS data do not support the use of this indicator to evaluate clinical outcomes or appropriateness of surgical treatments for breast cancer. Improvement of HIS coding accuracy and completeness could make it possible the use of administrative data for epidemiological purposes.

## Competing interests

The author(s) declare that there are no competing interests

## Authors' contributions

All authors participated in the design of the study, the definition of the statistical analysis and in the critical discussion of the results. All authors read and approved the final manuscript. PS conceived the study, contributed in performing the statistical analysis and drafted the manuscript. PP participated in conceiving the study, in the draft of the manuscripts and in performing the statistical analysis. NA, MS, PB and CAP defined the clinical aspects of the study design and interpreted the results from a clinical point of view.

## Pre-publication history

The pre-publication history for this paper can be accessed here:



## Figures and Tables

**Table 1:** Classification of breast cancer according to the severity indicator (SI)

**Categories**	**description**	**primary diagnosis**	**secondary diagnosis**
		*ICD9 -CM codes*	

**1**	I*n situ *tumors	233.0	-
**2**	Localized tumors	174.0 – 174.9	-
**3**	Non-localized tumors	174.0 – 174.9; 233.0	196
**4**	Metastatitc tumors	174.0 – 174.9; 233.0	197; 198

**Table 2:** Under and over-treatment categories used for the evaluation of appropriateness

	**pT**	**Surgery procedures**	**Linfoadenectomy**	**Stage**	**SI**
**Under-treatment**					
	T1 T2 T3	85.2*	no	I IIa IIb IIIa IIIb IV	2 3 4
Case a)	T1 T2 T3	85.41–85.42	no	I IIa IIb IIIa IIIb IV	2 3 4
	T2 T3	85.2*	yes	IIa IIb IIIa IIIb	3 4
	T4	85.2*, 85.41–85.44		IIIb	4

**Over-treatment**					
	Tis	85.4*	yes	0	1
	Tis	85.2*	yes	0	1
Case b)	T1 T2 T3	85.45–85.48		I IIa IIb IIIa IIIb	2 3
	T1§	85.41–85.44		I	2

§this case represents an over-treatment only if happens in more than 15% of the T1 cancers treated

Example:

a) case with tumor size T3, independently on the stage, who undergoes to intervention 85.41 (simple mastectomy) and no linfoadenectomy, is classified as under-treated

b) radical mastectomy (85.45–85.48) in a case with a tumor size less than T4, is always considered as an over-treatment

**Table 3:** Cases distribution according to the three classifications (pT, TNM, SI)

**pT**			**TNM**			**SI**		
	***n***	***%***		***n***	***%***		***n***	***%***

**Tis**	11	2.8	**0**	13	3.4	**1**	15	3.9
**T0**	2	0.5	**I**	146	37.7	**2**	257	66.4
**T1**	186	48.1	**II**	131	33.9	**3**	102	26.4
**T2**	97	25.1	**III**	40	10.3	**4**	2	0.5
**T3**	7	1.8	**IV**	4	1.0	*missing*	11	2.8
**T4**	31	8.0	*missing*	53	13.7	*missing*	53	13.7
**total**	**387**	**100**	**total**	**387**	**100**	**total**	**387**	**100**

**Table 4:** Agreement between SI – pT and TNM-pT for under- and over-treatment. Standard of reference: pT.

		**Under treatment**						
		**pT**						
			
		**yes**			**no**			**tot**
		n	col%	row%	n	col %	row%	
**SI**	**yes**	55	***58,5***	***67,1***	27	*11,3*	*32,9*	82
	**no**	39	*41,5*	*47,6*	213	***88,8***	*84,5*	252
	**tot**	94	*100,0*		240	*100,0*		334
		**pT**						
			
		**yes**			**no**			**tot**
		n	col%	row%	n	col %	row%	
**TNM**	**yes**	91	***96,8***	***77,8***	26	*10,8*	*22,2*	117
	**no**	3	*3,2*	*1,4*	214	***89,2***	*98,6*	217
	**tot**	94	*100,0*		240	*100,0*		334
	
		**Over treatment**						
		**pT**						
			
		**yes**			**no**			**tot**
		n	col%	row%	n	col %	row%	
**SI**	**yes**	15	***88,2***	***78,9***	4	*1,3*	*21,1*	19
	**no**	2	*11,8*	*0,6*	313	***98,7***	*99,4*	315
	**tot**	17	*100,0*		317	*100,0*		334
		**pT**						
		**yes**			**no**			**tot**
		n	col%	row%	n	col %	row%	
**TNM**	**yes**	17	***100,0***	***94,4***	1	*0,3*	*5,6*	18
	**no**	0	*0,0*	*0,0*	316	***99,7***	*100,0*	316
	**tot**	17	*100,0*		317	*100,0*		334

In bold are sensitivity (col% of yes), specificity (col% of no) and PPV (row% of yes)

**Table 5:** Validity of HIS data necessary to calculate the SI

		**Si calculated from HIS data**								
	
		**ca in situ**		**localized**		**not-localized**		**matastatic**		**total**
		
		*n*	*%*	*n*	*%*	*n*	*%*	*n*	*%*	*n*
		
**SI calculated from CC**	**ca in situ**	1	***11,1***	14	*4*	0	*0*	0	*0*	15
	**localized**	5	*57*	240	***75***	10	*24*	0	*0*	255
	**not-localized**	3	*33*	67	*21*	31	***76***	0	*0*	101
	**metastatic**	0	*0*	0	*0*	0	*0*	2	***100***	2
	
	**total**	9	*100*	321	*100*	41	*100*	2	*100*	373

Kappa= 0.35

p < 0.00

values in bold represent the positive predictive value
